# Submicroscopic placental infection by non-*falciparum Plasmodium* spp.

**DOI:** 10.1371/journal.pntd.0006279

**Published:** 2018-02-12

**Authors:** Justin Y. A. Doritchamou, Richard A. Akuffo, Azizath Moussiliou, Adrian J. F. Luty, Achille Massougbodji, Philippe Deloron, Nicaise G. Tuikue Ndam

**Affiliations:** 1 MERIT, Intitut de Recherche pour le Developpement—Université Paris Descartes, Sorbonne Paris Cité, Paris, France; 2 Centre d’Etude et de Recherche sur le Paludisme Associé à la Grossesse et à l’Enfance (CERPAGE), Faculté des Sciences de la Santé, Université d’Abomey-Calavi, Cotonou, Benin; 3 Noguchi Memorial Institute for Medical Research, University of Ghana, Accra, Ghana; University of Melbourne, AUSTRALIA

## Abstract

**Background:**

Among the *Plasmodium* species that infect humans, adverse effects of *P*. *falciparum* and *P*. *vivax* have been extensively studied and reported with respect to poor outcomes particularly in first time mothers and in pregnant women living in areas with unstable malaria transmission. Although, other non-*falciparum* malaria infections during pregnancy have sometimes been reported, little is known about the dynamics of these infections during pregnancy.

**Methods and findings:**

Using a quantitative PCR approach, blood samples collected from Beninese pregnant women during the first antenatal visit (ANV) and at delivery including placental blood were screened for *Plasmodium* spp. Risk factors associated with *Plasmodium spp*. infection during pregnancy were assessed as well as the relationships with pregnancy outcomes.

*P*. *falciparum* was the most prevalent *Plasmodium* species detected during pregnancy, irrespective either of parity, of age or of season during which the infection occurred. Although no *P*. *vivax* infections were detected in this cohort, *P*. *malariae* (9.2%) and *P*. *ovale* (5.8%) infections were observed in samples collected during the first ANV. These non-*falciparum* infections were also detected in maternal peripheral blood (1.3% for *P*. *malariae* and 1.2% for *P*. *ovale*) at delivery. Importantly, higher prevalence of *P*. *malariae* (5.5%) was observed in placental than peripheral blood while that of *P*. *ovale* was similar (1.8% in placental blood). Among the non-*falciparum* infected pregnant women with paired peripheral and placental samples, *P*. *malariae* infections in the placental blood was significantly higher than in the peripheral blood, suggesting a possible affinity of *P*. *malariae* for the placenta. However, no assoctiation of non-*falciparum* infections and the pregnancy outcomes was observed

**Conclusions:**

Overall this study provided insights into the molecular epidemiology of *Plasmodium* spp. infection during pregnancy, indicating placental infection by non-*falciparum Plasmodium* and the lack of association of these infections with adverse pregnancy outcomes.

## Author summary

*P*. *falciparum* and *P*. *vivax* infections during pregnancy have been extensively studied. Meanwhile, the dynamics of other non-*falciparum* malaria infections during pregnancy is not well understood. We investigated the prevalence of *Plasmodium* spp. in samples collected from Beninese women early in pregnancy and at delivery using a quantitative PCR approach. Factors associated with *Plasmodium* spp. infection during pregnancy were assessed. *P*. *falciparum* was the most prevalent *Plasmodium* species detected. *P*. *malariae* and *P*. *ovale* infections were also detected early in pregnancy, and in the maternal peripheral and placental blood at delivery. Noteworthily, the high prevalence of *P*. *malariae* in the placenta of pregnant women with paired peripheral and placental samples, suggests a placental affinity of *P*. *malariae* during pregnancy. However, association of non-*falciparum* infections with pregnancy outcomes was not observed. This study provided insights into the molecular epidemiology of *Plasmodium* spp. infection during pregnancy, and highlighted the underestimated prevalence of non-*falciparum* infections in pregnancy-related malaria.

## Introduction

*Plasmodium falciparum* is responsible for most cases of malaria during pregnancy and is linked with poor outcomes both among mothers and their babies [1,2]. Pregnant women are at heightened risk of infection despite pre-existing immunity acquired from previous exposures that protects against clinical malaria. This increased susceptibility to malaria during pregnancy has been related to the ability of *P*. *falciparum* infected erythrocytes to sequester in the placenta, inducing a placental inflammation that may lead to foetal growth alteration, stillbirth, and low birth weight (LBW) of the babies [3–5]. Futhermore, the proliferation of this new parasite phenotype can lead to severe maternal anemia [6,7].

Among the other *Plasmodium* parasites that infect humans (*P*. *malariae*, *P*. *ovale*, *P*. *knowlesi* and *P*. *vivax*), *P*. *vivax* has been reported in Asia and Latin America to have consequences during pregnancy, such as maternal anemia and LBW deliveries [8–12]. However, there is no evidence of *P*. *vivax* sequestration in the placenta [13]. The detection of *P*. *malariae* and *P*. *ovale* in pregnant women [14–16] has raised concerns about their possible involvement in pregnancy-associated malaria, although it is still unclear whether they display the placental tropism that *P*. *falciparum* does. Thus, geographical distribution of non-*falciparum* Plasmodia in West Africa, where *P*. *falciparum* is the most prevalent malaria species, and their involvement in the pathogenesis of pregnancy-associated malaria need to be documented. A PCR-based approach was used to better detect the non-*falciparum* malaria parasites due to their low parasite densities and the difficulty to accurately differentiate the species by morphological analysis using microscopy [17]. Only few studies reported the detection of non-*falciparum* malaria parasites in the placental blood [14,18,19].

Current efforts to prevent and control malaria during pregnancy using drug-based preventive treatment or vaccine strategies essentially target *P*. *falciparum* [20]. In this study, we investigated the prevalence and impact of *Plasmodium* spp. infection early in pregnancy (at first antenatal visit, before the introduction of prevention treatment), and at delivery in maternal peripheral and placental blood samples collected from a cohort of Beninese pregnant women across different malaria transmission seasons.

## Methods

### Ethics statement

The Strategies To Prevent Pregnancy Associated Malaria (STOPPAM) project was approved by the Comité Consultatif de Déontologie et d’Ethique of the Research Institute for Development (France) and the ethical committee of the Faculty of Health Science (University of Abomey-Calavi, Benin). All procedures complied with European and French national regulations. Written informed consent was given by participants who were all adults.

### Study samples and DNA extraction

Samples used in this study were collected from pregnant women during the STOPPAM study conducted from 2008 to 2010 in Southern Benin. Details of the project have been reported elsewhere [21]. Briefly, 1037 pregnant women with a gestational age under 24 weeks were enrolled early in pregnancy, during their first antenatal visit (ANC), and followed-up monthly till delivery. Upon admission to the study, pregnant women were scheduled for supervised intermittent preventive treatment (IPTp) doses of sulfadoxime pyrimethamine (SP) uptake and insecticide-treated nets (ITNs) were provided to them. Ultrasound scan was performed for gestational age determination. Hemoglobin (Hb) level was determined at each visit and birth weight was recorded at delivery. Peripheral and placental blood samples were collected from delivering women. Thick and thin blood films were prepared from all samples for microscopical detection of *Plasmodium* spp. The blood smears were Giemsa-stained in phosphate-buffered saline (PBS) and examined by two independent microscopists. A third read was performed in case of discrepancies between the first two reads. Two hundred microliters of blood pellet were stored at -20°C for DNA extraction.

Based on the available data of the parameters to be considered in this study, 975 peripheral blood samples collected at enrolment, and 667 peripheral and 562 placental blood samples collected at delivery were analysed. No placenta histology data was available for this study. Genomic DNA (gDNA) was extracted from the frozen whole blood using the DNAeasy Blood & Tissue kit (Qiagen), as recommended by the manufacturer.

### *Plasmodium* detection and quantification by real-time PCR

Species of *Plasmodium* spp. were detected in the whole blood DNA samples as described [22]. Briefly, a dual amplification was performed using *Plasmodium*-specific primers and probes previously published [23], and a detection primers/probes system for the human *GAPDH* gene GAPDH_Fw: CCTCCCGCTTCGCTCTCT, GAPDH_Rev: GCTGGCGACGCAAAAGA) and GapdhProbe: VIC-CCTCCTGTTCGACAGTCAGCCGC–MGBNFQ). *GAPDH* was used as an internal control gene to ensure that gDNA was successfully extracted. *Plasmodium* load was quantified by extrapolation of cycle thresholds (Ct) from a 6 fold standard curve of *Plasmodium* ring-infected erythrocytes. Samples without amplification (no Ct detected) were considered negative, and a density of 2 parasites/μl was assigned if amplification was observed out of the lower range of the standard curve (5 parasites/μl). A negative control with no DNA template was run in all reactions. This study benefited from a quality check program that was established to ensure correct performance of qPCR techniques between several laboratories including ours [22].

*Plasmodium* species (*P*. *falciparum*, *P*. *malariae* and *P*. *ovale*) were detected in a multiplex PCR reaction using species-specific forward and reverse primers, as well as specific probes, as described by Taylor et al. [22]. Specific primers targeting both *P*. *ovale curtisi* and *P*. *ovale wallikeri* were used to amplify both variants of *P*. *ovale* [24]. For *P*. *vivax* detection, samples were pooled into group of 10 samples and screened as single PCR reaction. Amplification was performed using *P*. *vivax* specific primers and probe under the same conditions described above with few modifications. A total of 45 cycles was performed, and samples from any pool with amplifications have been individually screened for *P*. *vivax* detection.

### Data analysis

Statistical analysis was conducted using STATA software version 13. Prevalence of *Plasmodium* spp. infection at different time-points of collection were determined according to the age and the parity of the pregnant women. The influence of the season and intensity of malaria transmission were explored in relation to *Plasmodium* spp. infections among pregnant women. Months in the year were coded from 1 to 12 respectively for January to December. Dry seasons were defined as December-March and August (12, 1, 2, 3 and 8) while wet seasons were defined as April-July (4–7) and Sep-Nov (9–11), as previously described [21]. *P*. *ovale* and *P*. *malariae* infections were combined and analyzed as non-*falciparum* infections. Infections involving *P*. *falciparum* and non-*falciparum* parasites together were grouped as mixed infections. Furthermore, a multi-variable logistic regression model was used to assess the risk factors for *P*. *falciparum*, non-*falciparum* and mixed infections at different time points by controlling for potential confounders and using those without malaria infection as the reference group. In addition, an exploratory analysis was conducted at selected time-points to determine whether there was any association between *Plasmodium* spp. infections and primary pregnancy outcomes (LBW, prematurity and maternal anaemia), and active PM. For this analysis, groups of women presenting each outcome were considered, and the distribution of *P*. *falciparum* and non-*falciparum* in the detected infections early in pregnancy and at delivery was investigated in comparison to women with no malaria infection from the corresponding outcome group. Active microscopic PM was defined as confirmed *Plasmodium* spp. infection in placental blood by microscopy, and was found to be exclusively *P*. *falciparum* active PM. All statistical tests were conducted at 0.05 level of significance.

## Results

### Description of *Plasmodium* spp. infections detected by microscopy and qPCR

Parasites density generated from qPCR amplification of *Plasmodium* spp. as well as the parasite load of the infections determined by microscopy, are reported in Table 1. All the infections detected by microscopy were positive by qPCR screening. However, more than 60% of the infections detected by qPCR (61% at enrolment, 71% of peripheral and 63% of placental infections at delivery) have been missed by microscopy. It is important to note that none of the submicroscopic infections were associated with fever, suggesting an asymptomatic infection.

**Table 1:** Parasite load of infections detected by microscopy and PCR.

	Inclusion	Delivery	
		Peripheral	Placental
**Microscopy**			
**Number**	162	70	73
**Mean ± SD**	1593.94 ± 4115.59	15265.89 ± 38755.36	23367.85 ± 73418.04
**Median (IQR)**	311 (153–1456)	1142.5 (301–6957)	1268 (287–8552)
**Min—Max**	24–35745	14–258389	46–522183
**PCR**			
**Number**	425	244	199
**Mean ± SD**	5629.71 ± 36441.53	13420.87 ± 76045.49	285903.2 ± 3850952
**Median (IQR)**	158.2 (24.71–1125.05)	12.23 (2–284.15)	26.98 (6.09–458.68)
**Min—Max**	2–1995045.6	2–846618.82	2–54328425.79

SD = Standard Deviation; IQR = Interquartiles; Min = minimum; Max = maximum. Placental parasitaemia by PCR are shown to be indicative, as they are over-estimated from mature forms.

### Prevalence of *Plasmodium* spp. infection early in pregnancy

Among samples collected early in pregnancy at the inclusion time-point, *P*. *falciparum* was the most prevalent *Plasmodium* species found in infected women. *P*. *falciparum* infection was detected in 39.3% of samples analysed either as a mono-species infection (29.6%) or involved in mixed infection (9.7%) with other *Plasmodium* species (Table 2). Infections involving *P*. *malariae* and *P*. *ovale* were detected respectively in 9.2% and 5.7% of samples. It appeared that infections with *P*. *malariae* early in pregnancy were more prevalent than those involving *P*. *ovale* (Chi square test, p = 0.001). When the analysis was performed considering the non-*falciparum Plasmodium* parasites as mono-species infection, a reverse trend was observed. Indeed, the prevalence of *P*. *malariae* mono-infection (1.4%) was significantly lower as compared to *P*. *ovale* mono-infection (2.3%) (Chi square test, p < 0.0001). Mixed infections involving *P*. *falciparum* and non-*falciparum* were detected in 98 (10.1%) samples as reported in Table 2. Infection with *P*. *vivax* was not detected in samples analysed in this work.

**Table 2:** *Plasmodium* spp. infections in Beninese pregnant women.

	Inclusion	Delivery	
		Peripheral	Placental
**Number of pregnant women**	975	667	562
**Number of *P*. *falciparum* infections**	383	231	180
Prevalence *(95% CI)*	39.3% (36.3–42.4)	34.6% (31.1–38.3)	32.0% (28.3–36.0)
**Number of *P*. *malariae* infections**	90	9	31
Prevalence (95% CI)	9.2% (7.6–11.2)	1.3% (0.7–2.6)	5.5% (3.9–7.7)
**Number of *P*. *ovale* infections**	56	8	10
Prevalence (95% CI)	5.7% (4.4–7.4)	1.2% (0.6–2.4)	1.8% (1.0–3.3)
**Mono-infections, no. (%)**			
*P*. *falciparum*	289 (29.6)	227 (34.0)	162 (28.8)
*P*. *malariae*	14 (1.4)	5 (0.7)	15 (2.7)
*P*. *ovale*	22 (2.3)	8 (1.2)	2 (0.4)
**Mixed infections, no. (%)**			
*P*. *falciparum*, *P*. *malariae*	64 (6.6)	4 (0.6)	12 (2.1)
*P*. *falciparum*, *P*. *ovale*	22 (2.3)	0 (0)	4 (0.7)
*P*. *falciparum*, *P*. *malariae*, *P*. *ovale*	8 (0.8)	0 (0)	2 (0.4)
*P*. *malariae*, *P*. *ovale*	4 (0.4)	0 (0)	2 (0.4)

### Relation of parity and age with *P*. *falciparum* and non-*falciparum* infections early in pregnancy

The prevalence of *Plasmodium* spp. infections at enrolment was analysed according to the parity of the women and their age (Table 3). The prevalence of *P*. *malariae* and *P*. *ovale* infection was combined as non-*falciparum* and *P*. *falciparum* infections were analysed as mono-infection while mixed infections were considered as a separate group. *P*. *falciparum* infection occurring early in pregnancy was significantly associated with parity (Chi square test, p = 0.005) and age (Chi square test, p < 0.0001), resulting in higher prevalence of *P*. *falciparum* infection in primigravid and younger women. No relation was observed when non-*falciparum* infections at enrolment were compared with parity and age of the women.

**Table 3:** Prevalence of *Plasmodium spp*. infections during pregnancy according to parity and age of the woman, and the season of sample collection.

	Inclusion, no. (%)							Peripheral Blood, no. (%)						Placental Blood, no. (%)					
	*Pf*	P *	non*-Pf*	P *	Mixed		P *	*Pf*	P *	non*-Pf*	P *	Mixed	P *	*Pf*	P *	non*-Pf*	P *	Mixed	P *
**Parity**		0.005		0.75		0.17			0.92		0.55		0.08		0.20		0.98		0.94
**Primiparae**	68 (38.4)		8 (4.5)		22 (12.4)			38 (33.6)		3 (2.7)		2 (1.8)		31 (34.4)		3 (3.3)		3 (3.3)	
**Multiparae**	221 (27.7)		32 (4.0)		72 (9.0)			189 (34.1)		10 (1.8)		2 (0.4)		131 (27.8)		16 (3.4)		15 (3.2)	
**Age**		0.000		0.60		0.49			0.34		0.73		0.000		0.02		0.35		0.92
**< 18 years**	16 (43.2)		3 (8.1)		4 (10.8)			11 (39.3)		1 (3.6)		2 (7.1)		11 (50.0)		0 (0.0)		1 (4.6)	
**18–20 years**	75 (42.6)		8 (4.5)		22 (12.5)			45 (38.8)		3 (2.6)		0 (0.0)		34 (34.0)		6 (6.0)		3 (3.0)	
**21–24 years**	53 (29.6)		7 (3.9)		18 (10.1)			32 (28.1)		1 (0.9)		0 (0.0)		20 (21.1)		2 (2.1)		4 (4.2)	
**25 years+**	139 (24.4)		21 (3.7)		49 (8.6)			134 (33.8)		8 (2.0)		2 (0.51)		91 (27.3)		11 (3.3)		10 (3.0)	
Season**		0.47		0.75		0.04			0.51		0.80		0.31		0.43		0.008		0.12
**Other months**	119 (30.6)		17 (4.4)		38 (9.8)			107 (32.4)		7 (2.1)		3 (0.9)		70 (26.3)		15 (5.6)		5 (1.9)	
**April-July**	50 (26.0)		6 (3.1)		10 (5.2)			84 (34.9)		5 (2.1)		0 (0.0)		67 (31.5)		1 (0.5)		11 5.2)	
**Sep-Nov**	20 (30.5)		17 (4.3)		46 (11.7)			36 (38.7)		1 (1.1)		1 (1.1)		25 (30.9)		3 (3.7)		2 (2.5)	

*Pf* **=** *Plasmodium falciparum*; non-*Pf* = non-*falciparum*

*P value

**Season of enrolment and season of delivery for peripheral and placental blood

### Sub-microscopic detection of non-*falciparum* infection at delivery

*Plasmodium* spp. infection at delivery was assessed in the peripheral and placental blood samples. *P*. *falciparum* was, by far, the most prevalent *Plasmodium* species detected in maternal peripheral (34.6%) and placental blood (32%) samples (Table 2). *Plasmodium* spp. infections involving *P*. *malariae* and *P*. *ovale* at delivery were detected respectively in 1.3% and 1.2% of maternal peripheral blood samples, resulting in non significant difference in the prevalence of both parasites in the maternal peripheral blood samples at delivery. Only *P*. *falciparum* and *P*. *malariae* mixed infections were detected in 4 out of 667 maternal peripheral blood samples. Remarkably, in the placental samples, the prevalence of *P*. *malariae* infections (5.5%) was significantly higher than those involving *P*. *ovale* (1.8%) (Chi square test, p < 0.0001). Mixed infections comprising *P*. *falciparum*/*P*. *malariae*; *P*. *falciparum*/*P*. *ovale*; *P*. *falciparum*/*P*. *malariae*/*P*. *ovale* and *P*. *malariae*/*P*. *ovale* were respectively detected in 12 (2.1%); 4 (0.7%); 2 (0.4%) and 2 (0.4%) placental samples. Most importantly, mono-infections with *P*. *malariae* were detected in the placenta of 15 women (2.7%), at a higher prevalence compared to that of *P*. *ovale* (0.4%) (Chi square test, p < 0.0001). However, this difference was not observed in the maternal peripheral samples (0.7% for *P*. *malariae* and 1.2% for *P*. *ovale*). As at enrolment, no *P*. *vivax* infection was detected in the samples collected at delivery.

In the maternal peripheral blood samples at delivery, neither *P*. *falciparum* nor non-*falciparum* infections were associated with parity or age of the women. However, younger mothers were the most exposed to mixed infections (p < 0.001), while no difference was observed in the prevalence of *P*. *falciparum* and non-*falciparum* infections over different age groups and the parity of the women (Table 3).

*P*. *falciparum* infection in placental blood samples was significantly associated with the women’s age (p = 0.025), suggesting that younger pregnant women are more susceptible to develop a placental *P*. *falciparum*-malaria although being co-infected with other *Plasmodium* species. It is worth noting that although not statistically significant, primigravid women had higher prevalence of *P*. *falciparum* than multigravid women.

### Influence of seasons on *Plasmodium* spp. infection during pregnancy

**Fig 1:**
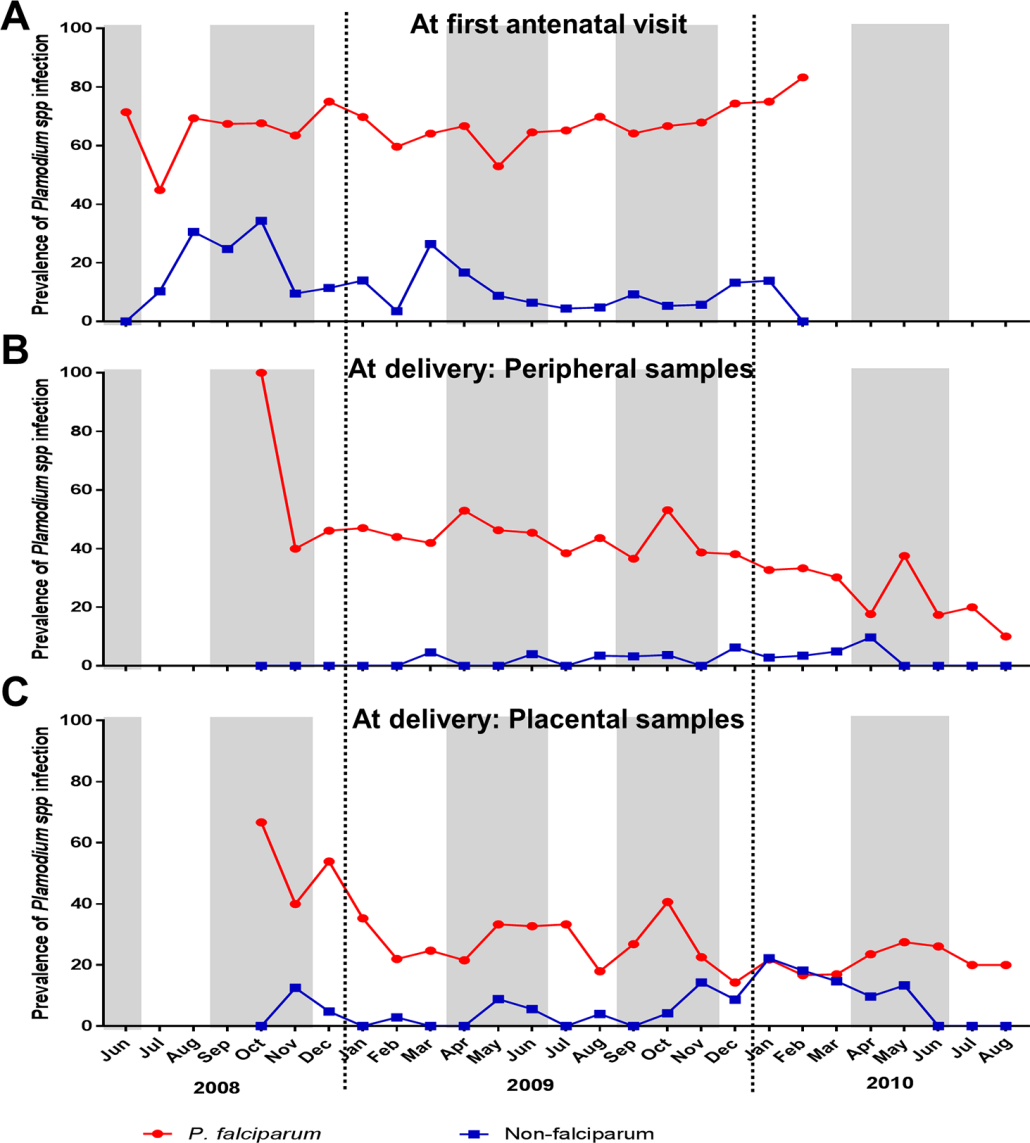
Monthly prevalence of *P*. *falciparum* and non-*falciparum* infections in Beninese pregnant women. The prevalence of *P*. *falciparum* (in red) and non-*falciparum* (in blue) infections detected in pregnant at the enrollment (A) and at delivery in the peripheral (B) and placental (C) blood samples are presented. Periods covered June 2008 to February 2010 corresponding to the recruitment of the pregnant women and October 2008 to August 2010 for the data collection at delivery.

The prevalence of *P*. *falciparum* and non-*falciparum* infections was analysed according to seasons corresponding to the blood sampling. The monthly prevalences of *P*. *falciparum* and non-*falciparum* infections at enrolment and at delivery are shown in Fig 1. Over the period of sample collection in early pregnancy stage, the monthly prevalence of *P*. *falciparum* infections (range, 45%–84%) was significantly higher than non-*falciparum* infections (range, 0%–34%) (Wilcoxon matched-pairs signed rank test, p < 0.0001). This differential monthly prevalence of *P*. *falciparum* and non-*falciparum* infections was maintained at delivery with a high prevalence of *P*. *falciparum* infections in peripheral and placental blood samples (Wilcoxon matched-pairs signed rank test, p < 0.0001 for both peripheral and placental samples). However, over three successive months (January to March 2010), similar prevalence values of *P*. *falciparum* and non-*falciparum* placental infections were observed. Of note, *Plasmodium* spp. infection early in pregnancy clearly appeared to be high regardless of season and irrespective to the *Plasmodium* species. In these samples collected at enrolment, no distinct pattern of overlapping peaks of *Plasmodium* spp. prevalence and the rainy seasons was observed, although a significant increase of mixed infections (p = 0.045) was detected between September and November (Table 3). However, season was not a risk factor for *Plasmodium* spp. infection at enrolment (Tables 4 and 5, S1 Table).

**Table 4:** Risk factors for *P*. *falciparum* malaria infections at enrolment.

Risk factors	Crude OR	p value	*Adjusted OR	p value
			(95% CI)	
**Gravidity**				
**Primiparae**	[Reference]	-	[Reference]	-
**Multiparae**	0.61 (0.44–0.86)	0.005	0.99 (0.65–1.52)	0.968
**Gestational age**				
**1st trimester (<13 weeks)**	[Reference]	-	[Reference]	-
**2nd trimester (13–26 weeks)**	1.37 (0.98–1.92)	0.069	1.23 (0.88–1.75)	0.228
**3rd trimester (>26 weeks)**	1	-	1	-
**Age**				
**< 18 years**	[Reference]	-	[Reference]	-
**18–20 years**	0.97 (0.48–1.99)	0.944	1.00 (0.48–2.11)	0.995
**21–24 years**	0.55 (0.27–1.14)	0.108	0.58 (0.27–1.27)	0.173
**25 years+**	0.42 (0.22–0.84)	0.013	0.45 (0.21–0.98)	0.044
**Season of enrolment**				
**Other months**	[Reference]	-	[Reference]	-
**April-July**	0.80 (0.54–1.18)	0.257	0.84 (0.56–1.25)	0.396
**Sep-Nov**	0.99 (0.73–1.35)	0.967	1.02 (0.74–1.39)	0.913

OR = Odd ratio

*Crude ORs for parity, gestational age, season of enrolment are adjusted for all other Covariates (parity, gestational age, mother’s age, season of enrolment).

**Table 5:** Risk factors for *P*. *falciparum* and non-*falciparum* malaria mixed infections at enrolment.

Risk factors	Crude OR	p vlaue	*Adjusted OR	p value
			(95% CI)	
**Gravidity**				
**Primiparae**	[Reference]	-	[Reference]	-
**Multiparae**	0.70 (0.42–1.16)	0.167	0.80 (0.42–1.51)	0.491
**Gestational age**				
**1st trimester (<13 weeks)**	[Reference]	-	[Reference]	-
**2nd trimester (13–26 weeks)**	1.02 (0.61–1.70)	0.934	0.96 (0.57–1.61)	0.873
**3rd trimester (>26 weeks)**	9.71 (1.30–72.56)	0.027	8.99 (1.18–68.17)	0.034
**Age**				
**< 18 years**	[Reference]	-	[Reference]	-
**18–20 years**	1.18 (0.38–3.65)	0.776	1.35 (0.42–4.33)	0.616
**21–24 years**	0.92 (0.29–2.90)	0.89	1.05 (0.31–3.56)	0.933
**25 years+**	0.78 (0.26–2.29)	0.647	0.99 (0.29–3.36)	0.987
**Season of enrolment**				
**Other months**	[Reference]	-	[Reference]	-
**April-July**	0.51 (0.25–1.04)	0.065	0.52 (0.25–1.08)	0.080
**Sep-Nov**	1.22 (0.78–1.92)	0.389	1.20 (0.76–1.90)	0.445

OR = Odd ratio

*Crude ORs for parity, gestational age, season of enrolment are adjusted for all other Covariates (parity, gestational age, mother’s age, season of enrolment).

At delivery, no association between the prevalence of maternal peripheral *Plasmodium spp* or *P*. *falciparum* infections and season was observed (Table 6). Conversely, in placental blood, a significantly higher number of non-*falciparum* infections samples was detected during the dry season (p = 0.008, Table 3) as compared to the wet season, when the risk of non-*falciparum* infections in placenta blood was significantly higher (Adjusted OR, 0.07; p = 0.012, S2 Table), and these infections were mostly caused by *P*. *malariae* (S3 Table). Unlike early in pregnancy, most peaks in the monthly prevalence of *P*. *falciparum* and non-*falciparum* infection profiles in both peripheral and placental blood samples at delivery matched with the rainy seasons (Fig 1).

**Table 6:** Risk factors for *P*. *falciparum* malaria infections at delivery.

	Delivery							
	Peripheral Blood				Placental Blood			
Risk factors	Crude OR	p value	*Adjusted OR (95% CI)	p value	Crude OR	p value	*Adjusted OR (95% CI)	p value
**Parity**	** **	** **	** **	** **	** **	** **	** **	** **
**Primiparae**	[Reference]	-	[Reference]	-	[Reference]	-	[Reference]	-
**Multiparae**	1.02 (0.67–1.57)	0.921	1.14 (0.65–2.00)	0.642	0.73 (0.45–1.18)	0.2	0.94 (0.49–1.81)	0.849
**Term at delivery**								
**Premature (<37 weeks)**	[Reference]	-	[Reference]	-	[Reference]	-	[Reference]	-
**Early term (37–38 weeks)**	0.75 (0.41–1.38)	0.361	0.72 (0.39–1.33)	0.290	1.60 (0.74–3.48)	0.234	1.67 (0.75–3.70)	0.208
**Full term (39–40 weeks)**	0.53 (0.31–0.92)	0.023	0.54 (0.31–0.94)	0.030	0.73 (0.35–1.52)	0.405	0.83 (0.39–1.76)	0.633
**Late term (41 weeks)**	0.76 (0.39–1.48)	0.42	0.79 (0.40–1.55)	0.488	0.68 (0.28–1.65)	0.388	0.74 (0.30–1.85	0.52
**Post term (> = 42 weeks)**	0.76(0.33–1.74)	0.519	0.81 (0.35–1.88)	0.620	1.30 (0.48–3.57)	0.607	1.34 (0.47–3.81)	0.589
**Age**								
**< 18 years**	[Reference]	-	[Reference]	-	[Reference]	-	[Reference]	-
**18–20 years**	0.98 (0.42–2.28)	0.962	0.92 (0.37–2.29)	0.852	0.52 (0.20–1.31)	0.163	0.62 (0.22–1.73)	0.362
**21–24 years**	0.60 (0.25–1.43)	0.25	0.57 (0.22–1.49)	0.252	0.27 (0.10–0.70)	0.008	0.31 (0.10–0.93)	0.037
**25 years+**	0.79 (0.36–1.74)	0.558	0.72 (0.28–1.88)	0.503	0.38 (0.16–0.90)	0.028	0.44 (0.15–1.32)	0.143
**Season of delivery**								
**Other months**	[Reference]	-	[Reference]	-	[Reference]	-	[Reference]	-
**April-July**	1.12 (0.78–1.58)	0.543	1.14 (0.80–1.63)	0.475	1.28 (0.86–1.91)	0.217	1.37 (0.91–2.07)	0.136
**Sep-Nov**	1.32 (0.82–2.12)	0.259	1.35 (0.83–2.20)	0.228	1.25 (0.72–2.16)	0.422	1.45 (0.82–2.56)	0.198

OR = Odd ratio

*Crude ORs for parity, gestational age, season of delivery are adjusted for all other Covariates (parity, gestational age, mother’s age, season of enrolment).

### Other risk factors of *Plasmodium* spp. infection early in pregnancy and at delivery

Factors associated with *P*. *falciparum* and non-*falciparum* malaria infection in pregnant women were assessed in samples collected early in pregnancy and at delivery. Parity has been identified as a risk factor of *P*. *falciparum* infection early in pregnancy. Indeed, relative to primigravid women, multigravid (crude OR, 0.52; p = 0.005) women had a significantly lower risk of *P*. *falciparum* infection early in pregnancy (Table 4). However, this parity-related effect disappeared when the analysis was adjusted with others covariates (Table 4). Interestingly, women older than 25 have lower risk of *P*. *falciparum* infection early in pregnancy (Adjusted OR, 0.45; p = 0.044). Parity, gestational age and age of the women did not significantly influence non-*falciparum* infections at enrolment, probably due to the limited size of this group (S1 Table). Strikingly, a greater risk of mixed infections was evident late in pregnancy, even though gestational age was not associated with *P*. *falciparum* and non-*falciparum* infections when analysed separately (Table 5, S1 Table).

At delivery, *P*. *falciparum* infection in maternal peripheral blood was a risk factor for premature delivery (Adjusted OR, 0.54; p = 0.03, Table 6). Women older than 21 presented a lower risk of *P*. *falciparum* placental infection than younger women.

### *Plasmodium* spp. infection and pregnancy outcomes

The relationships between *P*. *falciparum*, non-*falciparum* and mixed infection at enrolment and at delivery, and poor pregnancy outcomes such as prematurity, LBW and maternal anaemia at delivery were investigated (Tables 7 and 8). The proportion of women who developed each outcome from *P*. *falciparum*, non-*falciparum* and mixed infections groups was compared to the group of women with no malaria infection at delivery. Number of pregnant women with malaria infection in the placental blood determined by microscopy was significantly higher in women who were infected early in pregnancy with either non-*falciparum* or *P*. *falciparum* (p = 0.048 and p = 0.006, respectively), as compared to uninfected women at enrolment (Table 7). Noteworthily, only *P*. *falciparum* infections were significantly associated to maternal anemia at enrolment (S4 Table). No relationship was observed between non-*falciparum*, *P*. *falciparum* and mixed infections at enrolment, and the occurrence of LBW and maternal anemia at delivery. Nevertheless, mixed infection at enrolment was significantly related to premature delivery (p = 0.037), and a similar trend was found for *P*. *falciparum* infected mothers at enrolment and at delivery.

**Table 7:** Association of *Plasmodium* spp. infections at enrolment with pregnancy outcomes.

	No malaria, no. (%)*	Non-*falciparum*, no. (%)	P value**	*P*.* falciparum*, no. (%)	P value**	Mixed infection, no. (%)	P value**
**Active PM (n = 73)**	31 (9.0)	5 (21.7)	0.048	33 (17.1)	0.006	4 (6.9)	0.593
**Low birth weight (n = 102)**	52 (11.0)	4 (11.8)	0.893	37 (14.4)	0.177	9 (12.0)	0.802
**Anemia at delivery (n = 299)**	164 (43.6)	11 (45.8)	0.832	104 (50.7)	0.100	20 (35.1)	0.225
**Prematurity (n = 61)**	26 (5.7)	3 (8.8)	0.455	23 (9.2)	0.076	9 (12.2)	0.037

* Proportion of women who developed the corresponding outcome from each type of infection group is presented. Numbers for this analysis are detailed in S5 Table.

**P. *falciparum*, non-*falciparum* and mixed infections groups were compared with no malaria infection

**Table 8:** Association of *Plasmodium* spp. infections at delivery with pregnancy outcomes.

	No malaria, no. (%)*	Non-*falciparum*, no. (%)	P value**	*P*.* falciparum*, no. (%)	P value**	Mixed infection, no. (%)	P value**
** **	**PERIPHERAL BLOOD**						
**Active PM (n = 66)**	8 (2.2)	0 (0.0)	0.633	57 (29.1)	0.000	1 (33.3)	0.001
**Low birth weight (n = 69)**	39 (9.4)	1 (8.3)	0.897	28 (12.7)	0.208	1 (25.0)	0.293
**Anemia at delivery (n = 273)**	172 (44.2)	7 (53.9)	0.492	91 (45.5)	0.767	3 (75.0)	0.218
**Prematurity (n = 53)**	26 (6.3)	3 (8.8)	0.366	23 (10.7)	0.060	1 (25.0)	0.135
** **	**PLACENTAL BLOOD**						
**Active PM (n = 67)**	10 (2.8)	0 (0.0)	0.458	52 (32.1)	0.000	5 (27.8)	0.000
**Low birth weight (n = 56)**	36 (10.1)	1 (5.3)	0.488	18 (11.4)	0.670	1 (5.6)	0.525
**Anemia at delivery (n = 229)**	143 (42.8)	9 (50.0)	0.549	70 (47.3)	0.361	7 (43.8)	0.941
**Prematurity (n = 34)**	20 (5.7)	2 (10.5)	0.382	10 (6.4)	0.755	2 (11.1)	0.340

* Proportion of women who developed the corresponding outcome from each type of infection group is presented. Numbers for this analysis are detailed in S6 and S7 Tables.

** P. *falciparum*, non-*falciparum* and mixed infections groups were compared with no malaria infection

### Non-*falciparum* infections in paired peripheral and placental samples

Among pregnant women with non-*falciparum* infection at delivery, 38 (with *P*. *malariae* infection) and 18 (with *P*. *ovale* infection) paired peripheral and placental samples were analysed. *P*. *malariae* infections rate in the placental blood was significantly higher than in the peripheral samples (Fisher's exact test, p < 0.0001) (Table 9). A similar trend was observed when mono-infection with *P*. *malariae* was considered (Table 10). In a different way, distribution of *P*. *ovale* infections was similar in the peripheral and placental blood samples (S8 Table). However, when mono-infection with *P*. *ovale* was considered, more infections were detected in peripheral blood than in placental blood samples (Fisher's exact test, p < 0.0001) (S9 Table).

**Table 9:** Paired peripheral and placental samples of all *P*. *malariae* involved infections.

		Placental blood		Total (%)
		Negative	Positive	
**Peripheral blood**	**Negative**	0	29	29 (76.3)
	**Positive**	7	2	9 (23.7)
	**Total (%)**	7 (18.4)	31 (81.6)	

**Table 10:** Paired peripheral and placental samples of mono-infection of *P*. *malariae*.

		Placental blood		Total (%)
		Negative	Positive	
**Peripheral blood**	**Negative**	0	14	14 (73.7)
	**Positive**	4	1	5 (26.3)
	**Total (%)**	4 (21.1)	15 (79.0)	

## Discussion

The geographical distribution of non-*falciparum* malaria parasites is under re-evaluation due to continued development of more sensitive PCR-based diagnostic tools. Traditionally cited as uncommon causes of malaria in many African areas, *P*. *malariae*, *P*. *ovale* and *P*. *vivax* infections have been reported at increasing prevalences [25–29]. Common features of non-*falciparum* infections include chronicity, low parasite density, asymptomatic and multiple species infection [30]. In West Africa, these plasmodial infections have been detected in children [31,32], adults [32,33], and pregnant women [14–16]. However, the prevalence of non-*falciparum* infections among different populations remains undocumented in many West African countries, including Benin. We described the prevalence of different species of *Plasmodium* parasites that infect Beninese women during pregnancy, and confirm the presence of infections with *P*. *ovale* and *P*. *malariae* parasites among West African pregnant women, as previously reported [16]. Overall, *P*. *ovale* and *P*. *malariae* parasites were detected either as mono-infections, as dual infections or as mixed infections with *P*. *falciparum* in 3.7%, 0.4% and 9.3%, respectively, of pregnant women at first ANV, leading to a prevalence of non-*falciparum* malaria infection of 13.4%. This prevalence is higher than in other West African countries (Ghana, 0.96%; Mali, 3.81%; Gambia, 0.17% and Burkina Faso, 0.72%) [16], but close to that (9.4%) of Cameroonian pregnant women [14]. This high prevalence of non-*falciparum* infection might be explained by differences in study design and different epidemiological features, as well as by the highly sensitive PCR approach used [14,16].

No *P*. *vivax* infection was detected in pregnant women, although circulating *P*. *vivax* among non-pregnant Beninese was reported [34]. The authors who reported this presence of *P*. *vivax* selected a group based on sero-reactivity to r*Pv*MSP1 and r*Pv*CSP1 antigens [34], thus it is difficult to estimate the prevalence of *P*. *vivax* in a non-biased population.

Both at enrolment and delivery, *P*. *falciparum* was the most prevalent *Plasmodium* spp. supporting the general consensus that *P*. *falciparum* is responsible for most cases of pregnancy-related malaria. Indeed, *P*. *falciparum* infections either at enrolment or at delivery were related to active microscopically detectable PM, as reported [1,35]. At enrolment, prevalence of *P*. *falciparum* infection decreased with increasing age or parity, confirming the age- and parity-related susceptibility to malaria during pregnancy. Strikingly, this parity effect disappeared at delivery, suggesting a similar risk of *P*. *falciparum* infection for all pregnant women in the last weeks of pregnancy. This lack of parity-related susceptibility at delivery might be the effect of IPTp, which clears subsequent infections, but fails to protect against re-infections after the second dose of SP [36,37]. In fact, we have previously shown that uptake of IPT-SP doses in our cohort allowed to reduce the prevalence of malaria infections considerably, however re-infections were observed later in the follow-up, probably due to the decrease of the drug concentration in the blood over the time, as most of these re-infections were recrudescences [36]. An increased risk of placental infection when the first IPT-SP dose (and consequently, the second) was administered early in pregnancy [38] has been also demonstrated. These observations suggest that SP may significantly reduce parasite densities without clearing them up completely, probably also because of a level of parasite resistance to SP, even in West Africa where the mutant profiles differ from those in the East with a lower frequency of the quintuple mutants [36,39]. Our study was conducted at a time when Benin recommended two doses of SP for IPTp, that were initiated comparatively early in pregnancy (20–24 weeks gestational age), thus leaving a large part of the third trimester of pregnancy unprotected. Indeed, women were encouraged after the first ANV to use multiple malaria prevention tools, mainly IPTp and long-lasting insecticide treated nets, possibly obscuring the parity-related susceptibility of infection late in pregnancy. Furthermore, younger mothers still remained susceptible to *P*. *falciparum* placental infection which may persist from early pregnancy, since no age-related difference was observed in the prevalence of infection in maternal peripheral blood. This finding highlights the influence of IPTp, by reducing the prevalence of infection until delivery. On the other hand, this observation may also suggest that most *Plasmodium* spp. infections at delivery derived from new infections in IPTp-implemented areas.

A major feature of our data is the detailed description of non-*falciparum* malaria infection both early in pregnancy and at delivery, both in maternal peripheral and placental blood. In previous reports, *P*. *malariae* and *P*. *ovale* have been detected in placentas and placental blood samples [14,40] from pregnant women in Uganda and Cameroon. No evidence of sequestration in deep vascular beds was reported. This study revealed a relatively high prevalence of *P*. *malariae* and *P*. *ovale* in pregnant women at delivery, either as mixed or as mono-infections. The higher prevalence of *P*. *malariae* in the placental compartment compared with peripheral blood suggests a potential affinity of *P*. *malariae* for the placenta. This hypothesis requires further investigations to characterize *P*. *malariae* parasites, and to demonstrate any association with placental pathology.

Plasmodial infection during pregnancy occurs throughout the year in our study area where malaria transmission is continuous (Fig 1). Although season of enrolment was not a risk factor for *Plasmodium* spp. infection, the prevalence of mixed-*Plasmodium* infections was lower during the first rainy season. Similarly, a lower prevalence of non-*falciparum*, mainly *P*. *malariae*, was detected during both rainy seasons as compared to the dry season. At delivery, prevalence of non-*falciparum* placental blood infection was lower in the first rainy season. One limitation of this study is the lack of contemporary rainfall and entomological data at sample collection. Such information would allow to better define rainy and dry seasons, and to better correlate parasite prevalence and malaria transmission. This study showed that epidemiology of malaria early in pregnancy is not strictly dictated by transmission fluctuations, contrary to non-pregnant populations [41]. Some infections may simply emerge from asymptomatic (low-density) infections acquired early in pregnancy or before. Our findings contrast with reports from pregnant women in other areas where the rainy season was associated with an increased risk of malaria infection [42,43].

Many studies have demonstrated the role of *P*. *falciparum* in PM through the placental sequestration of *P*. *falciparum*-infected erythrocytes [44–46]. Here, non-*falciparum* and *P*. *falciparum*, but not mixed infections early in pregnancy were associated with placental blood infection at delivery. Women infected with non-*falciparum* at enrolment may be more susceptible to *P*. *falciparum* later in pregnancy, leading to PM at delivery. However, longitudinal studies are needed to investigate such putative role of early non-*falciparum* infection in promoting *P*. *falciparum* PM. Mixed infections at delivery displayed distinct relationships with gestational age, enrolment season, and age of the women. Although size differences of the groups may partly account for these observations, the contribution of non-*falciparum* parasites to the pathology and modulation of the immune response during co-infection with *P*. *falciparum* needs to be addressed.

## Supporting information

S1 Checklist STROBE checklist. (DOCX)

S1 Table Risk factors for non-*falciparum* malaria infections at enrolment. OR = Odd ratio; *Crude ORs for parity, gestational age, season of enrolment are adjusted for all other Covariates (parity, gestational age, mother’s age, season of enrolment)
(DOCX)

S2 Table Risk factors for non-*falciparum* malaria infections at delivery. OR = Odd ratio; *Crude ORs for parity, gestational age, season of delivery are adjusted for all other Covariates (parity, gestational age, mother’s age, season of enrolment).
(DOCX)

S3 Table Prevalence of *P*. *malariae* and *P*. *ovale* infections during the pregnancy according to parity and age of the woman, and the season of sample collection. *P value.
(DOCX)

S4 Table Association between Plasmodium spp infection and anemia at enrolment. * Proportion of women who developed the corresponding outcome from each type of infection group is presented. **P. *falciparum*, non-*falciparum* and mixed infections groups were compared with no malaria infection.
(DOCX)

S5 Table *Plasmodium* spp. infections at enrolment and pregnancy outcomes. (DOCX)

S6 Table *Plasmodium* spp. infections in the peripheral blood and pregnancy outcomes. (DOCX)

S7 Table *Plasmodium* spp. infections in the placental blood and pregnancy outcomes. (DOCX)

S8 Table Paired peripheral and placental samples of all *P*. *ovale* involved infections. (DOCX)

S9 Table Paired peripheral and placental samples of mono-infection of *P*. *ovale*. (DOCX)
