# Supplementary material for: Submicroscopic placental infection by non-falciparum Plasmodium spp
Source: PLoS Negl Trop Dis. 2018 Feb 12;12(2):e0006279. doi: 10.1371/journal.pntd.0006279 (PMC5825172; doi:10.1371/journal.pntd.0006279)
Supplement: S1 Table — OR = Odd ratio; *Crude ORs for parity, gestational age, season of enrolment are adjusted for all other Covariates (parity, gestational age, mother’s age, season of enrolment) (DOCX) [file pntd.0006279.s002.docx]

**S1 Table: Risk factors for non-*falciparum* malaria infections at enrolment**

| Risk factors | Crude OR | p value | *Adjusted OR (95% CI) | p value |
| --- | --- | --- | --- | --- |
| Gravidity |  |  |  |  |
| Primiparae | [Reference] | - | [Reference] | - |
| Multiparae | 0.88 (0.40-1.95) | 0.757 | 1.24 (0.45-3.46) | 0.674 |
| Gestational age |  |  |  |  |
| 1st trimester (<13 weeks) | [Reference] | - | [Reference] | - |
| 2nd trimester (13-26 weeks) | 1.22 (0.55-2.68) | 0.628 | 1.33(0.58-3.08) | 0.503 |
| 3rd trimester (>26 weeks) | 1 | - | 1 | - |
| Age |  |  |  |  |
| < 18 years | [Reference] | - | [Reference] | - |
| 18-20 years | 0.54 (0.14-2.14) | 0.380 | 0.49 (0.11-2.13) | 0.340 |
| 21-24 years | 0.46 (0.11-1.87) | 0.279 | 0.39 (0.08-1.89) | 0.245 |
| 25 years+ | 0.43 (0.12-1.53) | 0.194 | 0.36 (0.08-1.71) | 0.199 |
| Season of enrolment |  |  |  |  |
| Other months | [Reference] | - | [Reference] | - |
| April-July | 0.71 (0.27-1.82) | 0.471 | 0.74(0.28-1.91) | 0.529 |
| Sep-Nov | 0.99 (0.50-1.96) | 0.970 | 0.96(0.47-1.96) | 0.916 |

**Footnotes:** OR = Odd ratio; *Crude ORs for parity, gestational age, season of enrolment are adjusted for all other Covariates (parity, gestational age, mother’s age, season of enrolment)
