# Supplementary material for: Submicroscopic placental infection by non-falciparum Plasmodium spp
Source: PLoS Negl Trop Dis. 2018 Feb 12;12(2):e0006279. doi: 10.1371/journal.pntd.0006279 (PMC5825172; doi:10.1371/journal.pntd.0006279)
Supplement: S2 Table — OR = Odd ratio; *Crude ORs for parity, gestational age, season of delivery are adjusted for all other Covariates (parity, gestational age, mother’s age, season of enrolment). (DOCX) [file pntd.0006279.s003.docx]

**S2 Table: Risk factors for non-*falciparum* malaria infections at delivery**

|  | Peripheral Blood | | | | Placental Blood | | | |
| --- | --- | --- | --- | --- | --- | --- | --- | --- |
| Risk factors | **Crude OR** | **p** | ***Adjusted OR (95% CI)** | **p** | **Crude OR** | **p** | ***Adjusted OR (95% CI)** | **p** |
| Parity |  |  |  |  |  |  |  |  |
| Primiparae | [Reference] | - | [Reference] | - | [Reference] | - | [Reference] | - |
| Multiparae | 0.67 (0.18-2.49) | 0.55 | 0.654 (0.10-4.10) | 0.65 | 1.02 (0.29-3.57) | 0.03 | 1.09 (0.24-4.88) | 0.91 |
| Term at delivery |  |  |  |  |  |  |  |  |
| Premature (<37 weeks) | [Reference] | - | [Reference] | - | [Reference] | - | [Reference] | - |
| Early term (37-38 weeks) | 1.92 (0.21-17.56) | 0.56 | 2.06 (0.22-19.13) | 0.52 | 0.17 (0.01-1.87) | 0.15 | 0.16 (0.01-1.94) | 0.15 |
| Full term (39-40 weeks) | 1.27 (0.15-10.50) | 0.82 | 1.40 (0.17-11.80) | 0.76 | 0.61 (0.13-2.89) | 0.53 | 0.61 (0.12-3.12) | 0.55 |
| Late term (41 weeks) | 0.77 (0.05-12.48) | 0.85 | 0.75 (0.05-12.37) | 0.84 | 1.21 (0.21-6.95) | 0.83 | 1.12 (0.18-6.97) | 0.90 |
| Post term (>=42 weeks) | 1 | - | 1 | - | 1.32 (0.18-9.97) | 0.78 | 1.54 (0.19-12.76) | 0.69 |
| Age |  |  |  |  |  |  |  |  |
| < 18 years | [Reference] | - | [Reference] | - | [Reference] | - | [Reference] | - |
| 18-20 years | 0.72 (0.07-7.16) | 0.78 | 0.92 (0.08-10.95) | 0.95 | 1 | - | 1 | - |
| 21-24 years | 0.24 (0.01-3.94) | 0.32 | 0.33 (0.02-6.97) | 0.48 | 1.87 (0.67-5.19) | 0.23 | 1.82 (0.56-5.99) | 0.32 |
| 25 years+ | 0.56 (0.07-4.62) | 0.58 | 0.89 (0.06-14.11) | 0.93 | 0.63 (0.14-2.89) | 0.55 | 0.59 (0.12-3.00) | 0.53 |
| Season of delivery |  |  |  |  |  |  |  |  |
| Other months | [Reference] | - | [Reference] | - | [Reference] | - | [Reference] | - |
| April-July | 0.98 (0.31-3.12) | 0.97 | 0.89 (0.28-2.87) | 0.84 | 0.08 (0.01-0.60) | **0.01** | 0.07 (0.01-0.57) | **0.01** |
| Sep-Nov | 0.50 (0.06-4.13) | 0.52 | 0.50 (0.06-4.19) | 0.52 | 0.64 (0.18-2.28) | 0.49 | 0.59 (0.16-2.16) | 0.42 |

**Footnotes:** OR = Odd ratio; *Crude ORs for parity, gestational age, season of delivery are adjusted for all other Covariates (parity, gestational age, mother’s age, season of enrolment).
