# Supplementary material for: Submicroscopic placental infection by non-falciparum Plasmodium spp
Source: PLoS Negl Trop Dis. 2018 Feb 12;12(2):e0006279. doi: 10.1371/journal.pntd.0006279 (PMC5825172; doi:10.1371/journal.pntd.0006279)
Supplement: S3 Table — *P value. (DOCX) [file pntd.0006279.s004.docx]

**S3 Table: Prevalence of *P. malariae* and *P. ovale* infections during the pregnancy according to parity and age of the woman, and the season of sample collection**

|  | Inclusion, no (%) | | | | Peripheral Blood, no (%) | | | | Placental Blood, no (%) | | | | |
| --- | --- | --- | --- | --- | --- | --- | --- | --- | --- | --- | --- | --- | --- |
|  | ***P. malariae*** | **P *** | ***P. ovale*** | **P *** | ***P. malariae*** | **P *** | ***P. ovale*** | **P *** | ***P. malariae*** | **P *** | ***P. ovale*** | **P *** |  |
| Parity |  | 0.70 |  | 1.0 |  | 0.31 |  | 0.12 |  | 0.67 |  | 0.54 |  |
| Primiparae | 12 (1.5) |  | 18 (2.3) |  | 5 (0.9) |  | 5 (0.9) |  | 12 (2.5) |  | 2 (0.4) |  |  |
| Multiparae | 2 (1.1) |  | 4 (2.3) |  | 0 (0.0) |  | 3 (2.65) |  | 3 (3.3) |  | 0 (0.0) |  |  |
| Age |  | 0.60 |  | 0.59 |  | 0.70 |  | 0.61 |  | 0.15 |  | 0.73 |  |
| < 18 years | 1 (2.7) |  | 2 (5.4) |  | 0 (0.0) |  | 1 (3.6) |  | 0 (0.0) |  | 0 (0.0) |  |  |
| 18-20 years | 3 (1.7) |  | 4 (2.3) |  | 1 (0.9) |  | 2 (1.7) |  | 6 (6.0) |  | 0 (0.0) |  |  |
| 21-24 years | 4 (2.2) |  | 3 (1.7) |  | 0 (0.0) |  | 1 (0.9) |  | 2 (2.1) |  | 0 (0.0) |  |  |
| 25 years+ | 6 (111 |  | 13 (2.3) |  | 4 (1.0) |  | 4 (1.0) |  | 7 (2.1) |  | 2 (0.6) |  |  |
| Season |  | 0.17 |  | 0.38 |  | 0.74 |  | 0.46 |  | **0.02** |  | 0.28 |  |
| Other months | 9 (2.3) |  | 7 (1.8) |  | 3 (0.9) |  | 4 (1.2) |  | 12 (4.51) |  | 1 (0.4) |  |  |
| April-July | 2 (1.0) |  | 3 (1.6) |  | 1 (0.4) |  | 4 (1.7) |  | 1 (4.7) |  | 0 (0.0) |  |  |
| Sep-Nov | 3 (0.8) |  | 12 (3.1) |  | 1 (1.1) |  | 0 (0.0) |  | 2 (2.5) |  | 1 (1.2) |  |  |

**Footnote:** *P value
