# Supplementary material for: Submicroscopic placental infection by non-falciparum Plasmodium spp
Source: PLoS Negl Trop Dis. 2018 Feb 12;12(2):e0006279. doi: 10.1371/journal.pntd.0006279 (PMC5825172; doi:10.1371/journal.pntd.0006279)
Supplement: S4 Table — * Proportion of women who developed the corresponding outcome from each type of infection group is presented. **P. falciparum, non-falciparum and mixed infections groups were compared with no malaria infection. (DOCX) [file pntd.0006279.s005.docx]

**S4 Table: Association between Plasmodium spp infection and anemia at enrolment**

|  | No malaria, no. (%)* | Non-*falciparum*, no. (%) | P value** | | *P. falciparum*, no. (%) | | P value** | Mixed infection, no. (%) | P value** |
| --- | --- | --- | --- | --- | --- | --- | --- | --- | --- |
| Anemia at enrolment | 306 (55.8) | 21 (53.9) | | 0.809 | | 202 (70.4) | 0.000 | 63 (67.02) | 0.043 |

**Footnotes:** * Proportion of women who developed the corresponding outcome from each type of infection group is presented. ***P. falciparum*, non-*falciparum* and mixed infections groups were compared with no malaria infection
