# Supplementary material for: Submicroscopic placental infection by non-falciparum Plasmodium spp
Source: PLoS Negl Trop Dis. 2018 Feb 12;12(2):e0006279. doi: 10.1371/journal.pntd.0006279 (PMC5825172; doi:10.1371/journal.pntd.0006279)
Supplement: S5 Table — (DOCX) [file pntd.0006279.s006.docx]

**S5 Table: *Plasmodium spp.* infections at enrolment and pregnancy outcomes**

|  | No malaria, no. (%)* | Non-*falciparum*, no. (%) | *P. falciparum*, no. (%) | Mixed infection, no. (%) |
| --- | --- | --- | --- | --- |
| Active PM, no. | **343** | **23** | **193** | **58** |
| Negative | 312 (91.0) | 18 (78.3) | 160 (82.9) | 54 (93.1) |
| Positive | 31 (9.0) | 5 (21.7) | 33 (17.1) | 4 (6.9) |
| Low birth weight, no. | **472** | **34** | **256** | **75** |
| Negative | 420 (89.0) | 30 (88.2) | 219 (85.6) | 66 (88.0 |
| Positive | 52 (11.0) | 4 (11.8) | 37 (14.4) | 9 (12.0) |
| Anemia at delivery, no. | **376** | **24** | **205** | **57** |
| Negative | 212 (56.4) | 13 (54.2) | 101 (49.3) | 37 (64.9) |
| Positive | 164 (43.6) | 11 (45.8) | 104 (50.7) | 20 (35.1) |
| Prematurity, no. | **457** | **34** | **249** | **74** |
| No | 431 (94.3) | 31 (91.2) | 226 (90.8) | 65 (87.8) |
| Yes | 26 (5.7) | 3 (8.8) | 23 (9.2) | 9 (12.2) |
