# Supplementary material for: Submicroscopic placental infection by non-falciparum Plasmodium spp
Source: PLoS Negl Trop Dis. 2018 Feb 12;12(2):e0006279. doi: 10.1371/journal.pntd.0006279 (PMC5825172; doi:10.1371/journal.pntd.0006279)
Supplement: S6 Table — (DOCX) [file pntd.0006279.s007.docx]

**S6 Table: *Plasmodium spp.* infections in the peripheral blood and pregnancy outcomes**

|  | No malaria, no. (%)* | Non-*falciparum*, no. (%) | *P. falciparum*, no. (%) | Mixed infection, no. (%) |
| --- | --- | --- | --- | --- |
| Active PM, no. | **358** | **10** | **193** | **3** |
| Negative | 350 (97.8) | 10 (100.0) | 139 (70.9) | 2 (66.7) |
| Positive | 8 (2.2) | 0 (0.0) | 57 (29.1) | 1 (33.3) |
| Low birth weight, no. | **413** | **12** | **221** | **4** |
| Negative | 374 (90.6) | 11(91.7) | 193 (87.3) | 3 (75.0) |
| Positive | 39 (9.4) | 1 (8.3) | 28 (12.7) | 1 (25.0) |
| Anemia at delivery, no. | **389** | **13** | **200** | **4** |
| Negative | 217 (55.8) | 6 (46.2) | 109 (54.5) | 1 (25.0) |
| Positive | 172 (44.2) | 7 (53.9) | 91 (45.5) | 3 (75.0) |
| Prematurity, no. | **407** | **12** | **216** | **4** |
| No | 381 (93.6) | 12 (100.0) | 193 (89.4) | 3 (75.0) |
| Yes | 26 (6.4) | 0 (0.0) | 23 (10.7) | 1 (25.0) |
