# Supplementary material for: Submicroscopic placental infection by non-falciparum Plasmodium spp
Source: PLoS Negl Trop Dis. 2018 Feb 12;12(2):e0006279. doi: 10.1371/journal.pntd.0006279 (PMC5825172; doi:10.1371/journal.pntd.0006279)
Supplement: S7 Table — (DOCX) [file pntd.0006279.s008.docx]

**S7 Table: *Plasmodium spp.* infections in the placental blood and pregnancy outcomes**

|  | No malaria, no. (%)* | Non-*falciparum*, no. (%) | *P. falciparum*, no. (%) | Mixed infection, no. (%) |
| --- | --- | --- | --- | --- |
| Active PM, no. | **355** | **19** | **162** | **18** |
| Negative | 345 (97.2) | 19 (100.0) | 110 (67.9) | 13 (72.2) |
| Positive | 10 (2.8) | 0 (0.0) | 52 (32.1) | 5 (27.8) |
| Low birth weight, no. | **355** | **19** | **158** | **355** |
| Negative | 319 (89.9) | 18 (94.7) | 140 (88.6) | 319 (89.9) |
| Positive | 36 (10.1) | 1 (5.3) | 18 (11.4) | 36 (10.1) |
| Anemia at delivery, no. | **334** | **18** | **148** | **16** |
| Negative | 191 (57.2) | 9 (50.0) | 78 (52.7) | 9 (56.3) |
| Positive | 143 (42.8) | 9 (50.0) | 70 (47.3) | 7 (43.8) |
| Prematurity, no. | **353** | **19** | **157** | **18** |
| No | 333 (94.3) | 17 (89.5) | 147 (93.6) | 16 (88.9) |
| Yes | 20 (5.7) | 2 (10.5) | 10 (6.4) | 2 (11.1) |
