# Supplementary material for: Submicroscopic placental infection by non-falciparum Plasmodium spp
Source: PLoS Negl Trop Dis. 2018 Feb 12;12(2):e0006279. doi: 10.1371/journal.pntd.0006279 (PMC5825172; doi:10.1371/journal.pntd.0006279)
Supplement: S9 Table — (DOCX) [file pntd.0006279.s010.docx]

**S9 Table: Paired peripheral and placental samples of mono-infection of *P. ovale***

|  |  | **Placental blood** | | **Total (%)** |
| --- | --- | --- | --- | --- |
|  |  | **Negative** | **Positive** |  |
| **Peripheral blood** | **Negative** | 0 | 2 | 2 (20.0) |
|  | **Positive** | 8 | 0 | 8 (80.0) |
|  | **Total (%)** | 8 (80.0) | 2 (20.0) |  |
